# Retraction: Efficacy and safety of aripiprazole or bupropion augmentation and switching in patients with treatment-resistant depression or major depressive disorder: A systematic review and meta-analysis of randomized controlled trials

**DOI:** 10.1371/journal.pone.0357331

**Published:** 2026-09-01

**Authors:** The PLOS One Editors

Following the publication of this article [1], concerns were raised about the methodology. Specifically, three [2–4] of the five studies in the systematic review and meta-analysis include the same 1,522 patients with major depressive disorder, violating the assumption of independence.

PLOS independently confirmed that [2] is the main outcome study and presents data from the initial 12-week acute treatment period (the primary outcomes), [3] focuses on a different set of outcomes, measuring cost-effectiveness in the same patient group, and [4] focuses on the 24-week follow-up period after the acute treatment phase (secondary and exploratory outcomes). Furthermore, [3] appears not to have met the inclusion criteria set by the authors [1]. Treating three publications from the same underlying trial as three separate studies in the analysis inflates the number of independent studies behind the pooled result. This is especially relevant given that only five total studies were included. As a result, this may make the findings look more precise than they are and may give the shared patient cohort disproportionate weight in the pooled estimate compared with the two other studies.

The authors did not respond to editorial requests for comment on these concerns.

The *PLOS One* Editors sought advice from a Statistical Advisor with expertise in systematic reviews and meta-analyses regarding the concerns outlined above. They concluded that the concerns are valid. In light of the above concerns that question the validity of the published results and conclusions in [1], the *PLOS One* Editors retract this article.

All authors either did not respond directly or could not be reached.
